# Re: Simulation analysis for tumor radiotherapy based on three‐component mathematical models

**DOI:** 10.1002/acm2.12608

**Published:** 2019-05-30

**Authors:** Meghan C. Ferrall‐Fairbanks, Daniel J. Glazar, Renee J. Brady, Gregory J. Kimmel, Mohammad U. Zahid, Philipp M. Altrock, Heiko Enderling

**Affiliations:** ^1^ Department of Integrated Mathematical Oncology H. Lee Moffitt Cancer Center & Research Institute Tampa FL USA

Dear Editor,

We were excited to read the recent article by Hong & Zhang “Simulation analysis for tumor radiotherapy based on three‐component mathematical models”.^1^ This is a topic that deserves much attention, as mathematical predictions of treatment response may ultimately help personalize radiation dose and dose fractionation.^2^, ^3^ Hong & Zhang conclude from simulation results of their model four findings: (a) that a three‐compartment model impacts radiotherapy efficacy and that three factors influence the outcome of simulated radiotherapy: (b) the proportion of quiescent tumor cells, (c) radiation dose per fraction, and (d) radiosensitivity in the form of the α/β ratio. Although intuitive and well‐supported by literature, we found that none of these conclusions were warranted by the presented results and analyses. In fact, we would like to point out selected discrepancies and inaccuracies with regards to these specific claims, in addition to several discrepancies in the mathematical formulation of their model.

1. Hong & Zhang discuss the importance of introducing quiescent cells as a third compartment into a classical two‐compartment mathematical model of dividing and nondividing cells. As quiescent cells have reduced radiosensitivity and can make up various proportions of a tumor, explicit consideration for these cells is motivated. However, a rigorous analysis of the three‐ vs two‐compartment tumor growth and radiation response model is not provided, which questions the validity of the conclusion that their approach is better. The only comparison appears to be between the three‐compartment model and classic Gompertzian tumor growth without a nonproliferating compartment.

It is important to discuss how to model quiescence in the Gompertzian tumor growth model. Hong & Zhang assume a constant rate of proliferating cells transitioning to quiescence and vice versa. This assumption implies that these transitions will occur with the same rates, regardless of tumor volume or tumor volume‐to‐carrying capacity ratio. In the Gompertzian model, tumor growth rate is modulated by the volume‐to‐carrying capacity ratio, and as tumors approach carrying capacity, their growth rate decreases, and vice versa. The transition between proliferating and quiescent compartments is intrinsically built into the Gompertzian model, and radiation response should be modeled with the understanding of the structure of the chosen model. Norton & Simon have used the Gompertzian model to simulate cancer treatment response proportionally to the tumor growth rate — a visualization of the proportion of proliferating cells within the tumor.^4^ Recent research proposed to use the tumor volume‐to‐carrying capacity ratio as a temporally dynamic measure of the proliferating and quiescent tumor subpopulations for radiotherapy, in a logistic model.^2^, ^5^

- Hong & Zhang claim to provide a calibrated model with “proper” parameters. Model parameterization is, however, unclear. Table 1 contains “partial parameters for the model” and refers to a bioRxiv preprint (the reference should have been updated to the highly cited final article published in PLoS Computational Biology^6^). These parameters, however, were calibrated to simulate *total* tumor volume using the Gompertzian model, without a quiescent cell or nondividing population. Based on experimental tumor volume data, no conclusions about a quiescent compartment can be drawn, and it is unclear how the parameters associated with the quiescent cell compartment (p_12_, p_21_, p_23_) were derived. Furthermore, the Hong & Zhang model should arrive at the same total tumor volume as the experiments in their model calibration, just with consideration of a subpopulation that is not proliferating. Having a larger tumor volume due to quiescent cells not contributing to tumor carrying capacity is flawed. Additionally, Hong & Zhang simulate tumors on the order of 3,000 cubic cm, which represents an unrealistically large tumor in a mouse that weighs around 30 grams.
- Hong & Zhang conclude that larger fractional doses provide better tumor control and rapid model convergence. However, it is unclear what model compartments are converging and what they are converging to. Neither the text, nor figures nor figure legends provide sufficient information to derive which model informed this “convergence” statement, and which of the various parameter sets in Table 1 were used. Rigorous model and parameter analysis are missing. Therefore, individual simulation results may be mere model artifacts.

**Table 1:** 

GM	Three‐component model
*a* = 0.56, *b* = 0.0719	*a* = 0.653, *b* = 0.0719 *P*_12_ = 0.1, *P*_21_ = 0.1, *P*_13_ = 0.05, *P*_23_ = 0.05, *η* = 0.2
*a* = 0.742, *b* = 0.0792	*a* = 0.837, *b* = 0.081 *P*_12_ = 0.1, *P*_21_ = 0.1, *P*_13_ = 0.05, *P*_23_ = 0.05, *η* = 0.2

The authors did, however, vary the initial volume of quiescent cells in the tumor population to investigate the impact of the quiescent population size on tumor growth and on treatment outcome. We must disagree with Hong & Zhang's claim that “ it can be concluded that the initial volume of quiescent cells impacts the process of radiotherapy first, then as soon as smaller the volume of quiescent cells is, the weaker the impact is [sic]”. The differences between the simulation results  appear minimal and arguably below signal‐to‐noise detection threshold. The authors provide no quantification of that difference to evaluate the suggested impact by these differences.

Hong & Zhang simulate treatment response using different α/β values to discuss the impact of radiosensitivity to resulting tumor volume. While the impact of radiosensitivity on volumetric response to radiation is well documented, the presented data do not show this. The α/β ratios are neither systematically varied, nor do the authors clarify and justify the selection of the ratios corresponding to the proliferating and quiescent cell populations in their model.

Finally, we feel the urgency to comment on the mathematical formulation of the three‐compartment model. First, there are discrepancies in the representation of the Gompertzian model. While the Gompertzian model is in correct mathematical form, the authors' interpretation of tumor volume, *T*, diverges from the usual unitless number (tumor volume divided by carrying capacity volume), which one can apply the natural logarithm to. Furthermore, the authors define the carrying capacity (*K*) as being dependent on the initial tumor volume (*T_0_*), which does not follow traditional applications of ecological and population‐based modeling. Additionally, and perhaps most crucially to the target audience, the implementation of the Linear‐Quadratic (LQ) model is not intuitive. Traditionally, the LQ model is described in terms of the dose, (*D*), dependent survival fraction, $\text{SF}\left(D\right)=e^{\alpha D-\beta D^{2}}$, which would be the ratio of *T*/*T*_0_ using the author's nomenclature. Most radiation modeling literature implements the effect of radiation in a piece‐wise fashion.^7^, ^8^, ^9^, ^10^, ^11^ The way in which radiation is modeled is mathematically incorrect, and disagrees with the shapes of the presented simulation curves.

In summary, we believe that the questions Hong & Zhang are exploring are interesting. However, due to the reasons presented in this letter, the authors' conclusions are not substantiated by the presented results. We feel that the manuscript is blurring the contributions that integrated mathematical oncology offers to radiation oncology.

## CONFLICT OF INTEREST

The authors have no conflict of interest to report.
